# Bevacizumab in ovarian cancer therapy: current advances, clinical challenges, and emerging strategies

**DOI:** 10.3389/fbioe.2025.1589841

**Published:** 2025-05-15

**Authors:** Mingyue Zhang, Jun Zhu, Yin Bao, Qiang Ao, Xiaoling Mao, Zhengzhou Qiu, Yiming Zhang, Yang Chen, Hong Zhu, Jun Gao

**Affiliations:** ^1^ School of Clinical Medicine, Jiangxi University of Chinese Medicine, Nanchang, China; ^2^ Department of Gynecologic Oncology, Jiangxi Cancer Hospital and Institute, Jiangxi Clinical Research Center for Cancer, The Second Affiliated Hospital of Nanchang Medical College, Nanchang, China; ^3^ Jiangxi Medical College, Nanchang University, Nanchang, China; ^4^ Liangzhu Laboratory, Zhejiang University Medical Center, Hangzhou, Zhejiang, China; ^5^ CAS Key Laboratory of Separation Science for Analytical Chemistry, Dalian Institute of Chemical Physics, Chinese Academy of Sciences, Dalian, China

**Keywords:** bevacizumab, ovarian cancer, tumor angiogenesis, drug resistance, nanodrug delivery

## Abstract

Bevacizumab targets the vascular endothelial growth factor signaling pathway, inhibiting tumor angiogenesis and reshaping the tumor microenvironment, positioning it as a cornerstone in ovarian cancer management. Its mechanisms of action include blocking VEGF-A-induced endothelial cell proliferation, promoting vascular normalization, alleviating hypoxic conditions, and reversing immunosuppression. Key phase III clinical trials, including GOG-0218, AURELIA, and PAOLA-1, have demonstrated that Bevacizumab significantly extends progression-free survival in the maintenance treatment of newly diagnosed advanced ovarian cancer, platinum-sensitive or resistant recurrent disease, and HRD-positive patients, with a median PFS of up to 37.2 months. However, its impact on overall survival remains limited, and challenges such as drug resistance, treatment-related toxicities, and high costs persist. Future advancements will hinge on multidisciplinary innovation, including dual-targeting approaches such as VEGF/Ang-2 bispecific antibodies, combination immunotherapies, intelligent nanodrug delivery systems, and AI-driven dynamic biomarker stratification. The use of biosimilars and adaptive platform trials offers promise in reducing costs and improving accessibility. These technological innovations mark a shift in ovarian cancer treatment from traditional chemotherapy to precision medicine, presenting new opportunities to improve long-term patient survival.

## 1 Introduction

OC, one of the most lethal malignancies of the female reproductive system, exhibits marked geographic heterogeneity and molecular complexity (Webb and Jordan, 2024; Qin and Chen, 2022). Incidence rates have declined in developed countries, potentially due to the widespread use of oral contraceptives (Arshadi et al., 2024), but rising trends are observed in Eastern Europe and parts of Asia, particularly among women under 50 years old (Meagher et al., 2024). Epithelial OC accounts for 85%–90% of cases, with high-grade serous carcinoma (HGSC) comprising 70%–80% (de Nonneville et al., 2024). Key molecular alterations in ovarian cancer (OC) include TP53 mutations (>90%) and germline BRCA1/2 mutations (Miwa et al., 2024), with emerging evidence suggesting an origin in the fallopian tube fimbriae, such as in serous tubal intraepithelial carcinoma (STIC) (Yen et al., 2024). Risk factors for OC include BRCA1/2 mutations, with carriers facing lifetime risks of 54% and 23%, respectively (Miwa et al., 2024), which are further heightened by Lynch syndrome (Kiser et al., 2024). Nulliparity, early menarche, and late menopause significantly increase the risk (Merritt et al., 2025), while multiparity and breastfeeding offer protective effects (Sasamoto et al., 2024). Environmental exposures such as asbestos, talcum powder, and high-cholesterol diets are also associated with increased risk (Mirabelli and Terracini, 2024). Effective management of OC remains a major challenge, as approximately 60%–70% of patients present with advanced-stage disease (III/IV) at diagnosis (Nelson et al., 2024). Screening methods like CA125 combined with ultrasound have shown limited sensitivity, reducing their usefulness to high-risk genetic groups (Ishizawa et al., 2024). Although platinum-based chemotherapy initially achieves response rates of roughly 80%, relapses occur in 80% of patients within 2–3 years, with HGSC frequently developing platinum resistance (Tuninetti et al., 2024; Li N. et al., 2017). Driving resistance Mechanisms include enhanced DNA repair pathways, immunosuppressive tumor microenvironment (TME), and metabolic reprogramming (Pourmasoumi et al., 2024).

Targeted therapies and immunotherapies hold promise but face ongoing challenges. PARP inhibitors, such as Olaparib, have demonstrated prolonged progression-free survival (PFS) in BRCA-mutated patients; however, resistance often emerges (Park et al., 2024). Clinical trials combining immune checkpoint inhibitors, like atezolizumab, with anti-angiogenic agents, such as Bevacizumab, reveal synergistic effects, although overall response rate (ORR) can remain modest (Kurtz et al., 2023). Chemotherapy-induced neurotoxicity, myelosuppression, and high treatment costs continue to impose substantial physical, psychological, and financial burdens (Nogueira-Rodrigues et al., 2024). To address these limitations, developing early diagnostic biomarkers, optimizing treatment regimens, and exploring novel therapeutic approaches are critical. Angiogenesis plays a pivotal role in OC progression, not only sustaining tumor growth but also altering the TME. The VEGF-VEGFR signaling axis is central to this process. Binding of VEGF to VEGFR-2 activates downstream PI3K/AKT and RAS/MAPK pathways, driving endothelial cell proliferation, migration, and survival while promoting neovascularization (Zhou et al., 2024; He et al., 2014). This angiogenic activity fosters tumor cell invasiveness through epithelial-mesenchymal transition (EMT), disrupts vascular endothelial integrity, and increases vascular permeability, contributing to ascites formation (Tang et al., 2024). VEGF-mediated VE-cadherin phosphorylation weakens endothelial junctions, exacerbating vascular leakage and fluid accumulation (Mohamed et al., 2024; Hu et al., 2024). Hypoxia within the TME further amplifies these processes by stabilizing hypoxia-inducible factor-1α (HIF-1α), which upregulates VEGF expression and perpetuates a feedback loop of hypoxia and angiogenesis (Deng et al., 2024). HIF-1α also suppresses T cell function by enhancing PD-L1 expression, while recruiting regulatory T cells (Tregs) and tumor-associated macrophages (TAMs) that promote immune evasion (Garlisi et al., 2024; Wu et al., 2023; Hu et al., 2020). Cancer-associated fibroblasts (CAFs) secrete extracellular matrix components that create a stromal barrier, obstructing drug delivery (Wang X. et al., 2021; Chen DQ. et al., 2024). Hypoxia-driven glycolysis produces lactic acid, which acidifies the microenvironment, inhibits immune cell function, and activates matrix metalloproteinases (MMPs) that remodel the vascular basement membrane (Gonzalez-Avila et al., 2023).

Bevacizumab, an anti-VEGF monoclonal antibody, directly targets VEGF signaling but faces challenges stemming from the heterogeneity of the TME (Zhou et al., 2024). Abnormal vascular architecture, the presence of CAFs, and immunosuppressive cells contribute to a TME that impairs immune cell infiltration and function, thereby reducing the efficacy of both anti-angiogenic and immunotherapeutic approaches (Duru et al., 2020; Qian et al., 2023; Vienot et al., 2022; Guo et al., 2024). Resistance to Bevacizumab often arises through activation of alternative pro-angiogenic pathways, such as Angiopoietin/Tie2, underscoring the necessity for multi-pathway targeting strategies (Leong and Kim, 2020; Liu J. et al., 2023; Gao et al., 2023). Furthermore, tumor-derived exosomes have been implicated in metastasis and drug resistance by transferring pro-angiogenic factors, highlighting the need for a deeper understanding of these dynamic microenvironmental interactions and their role in therapeutic resistance (Chen C. et al., 2024). Elevated VEGF levels in patients with OC are associated with advanced disease stage, chemotherapy resistance, and reduced overall survival (OS) (Yu et al., 2013; Trifanescu et al., 2023; Li et al., 2004). Patients with high tumor VEGF expression exhibit a median survival time approximately 30%–40% shorter than those with low expression levels (Yamamoto et al., 1997). Preclinical studies have demonstrated the efficacy of anti-angiogenic therapies, including Bevacizumab, in suppressing VEGFR-2 phosphorylation, reducing tumor vascular density, and alleviating ascites in mouse models. Combining Bevacizumab with chemotherapy has shown increased treatment sensitivity and a reduction in peritoneal metastasis, with short-term administration improving vascular function and enhancing drug delivery (Liu et al., 2025a). Clinical trials have further confirmed Bevacizumab’s therapeutic benefits. When used alongside carboplatin-paclitaxel as first-line treatment, Bevacizumab significantly extends PFS, although its effect on OS is less clear (Burger et al., 2011). Additional studies, such as OCEANS and AURELIA, have validated Bevacizumab’s effectiveness in both platinum-sensitive and platinum-resistant recurrent cases (Aghajanian et al., 2012; Pujade-Lauraine et al., 2014). Moreover, combining Bevacizumab with PARP inhibitors or immune checkpoint inhibitors has demonstrated synergistic anti-tumor effects, reinforcing the value of combination strategies in improving outcomes (Guo et al., 2018; Jin et al., 2022; Monk et al., 2016).

Recently, Mao et al. (2022) and Rossi et al. (2017) have comprehensively summarized the mechanistic basis of bevacizumab and its limitations in early-phase clinical trials. While Liu S. et al. (2021) focused on its integration with conventional chemotherapy, and Yoshida et al. (2015) critically evaluated its clinical heterogeneity, emerging paradigms, such as nanotechnology-enabled drug delivery, AI-driven biomarker stratification, and dual-targeting strategies like VEGF/Ang-2 bispecific antibodies, remain underexplored. This review aims to bridge these gaps by synthesizing data from recent Phase III trials, such as PAOLA-1 and DUO-O, and proposing a multidisciplinary framework to address resistance mechanisms and advance precision oncology. Furthermore, we outline novel combination strategies, unresolved challenges, and future directions for optimizing bevacizumab-based regimens in ovarian cancer.

## 2 The role of bevacizumab in OC treatment

### 2.1 Bevacizumab’s mechanism of action in OC

**FIGURE 1:**
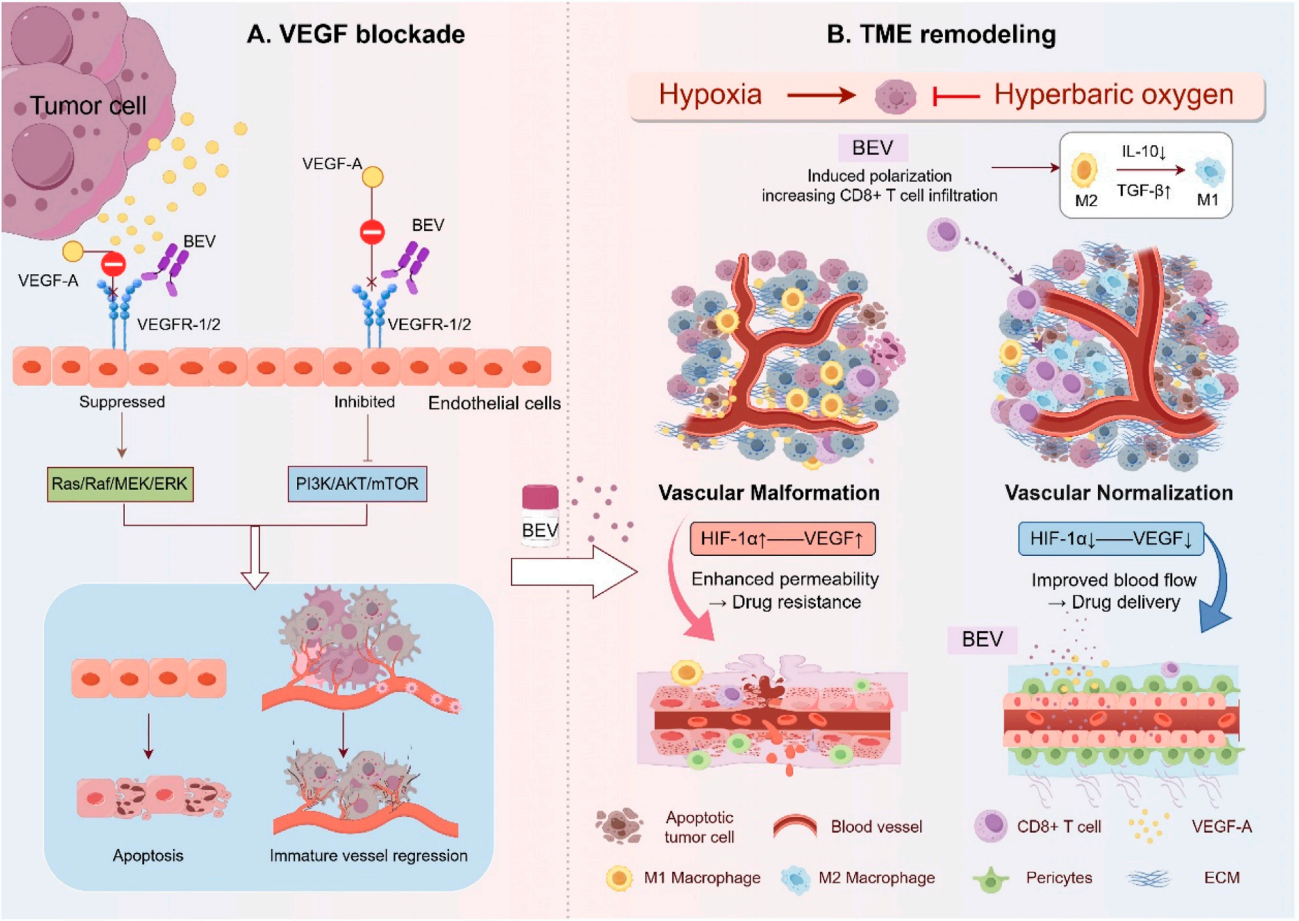
Mechanism of Bevacizumab in OC: VEGF Signaling Blockade and TME Remodeling. **(A)** Bevacizumab binds VEGF-A, preventing its interaction with VEGFR-1/2 on endothelial cells. This inhibits downstream pro-angiogenic signaling (Ras/ERK and PI3K/AKT pathways), resulting in endothelial apoptosis and regression of immature blood vessels. **(B)** In the TME, Bevacizumab induces vascular normalization by enhancing tight endothelial junctions and pericyte coverage, reducing hypoxia and acidosis, and reprograming immunosuppressive components, including converting M2 macrophages to M1 phenotypes and increasing CD8^+^ T cell infiltration. These changes improve chemotherapy delivery and anti-tumor immunity. This figure was created by Figdraw (https://www.figdraw.com).

Bevacizumab is a recombinant humanized IgG1 monoclonal antibody that primarily targets VEGF-A, particularly the VEGF isoform, to block the angiogenesis signaling pathway (Wang et al., 2004). By inhibiting VEGF-A, Bevacizumab effectively disrupts the process of tumor angiogenesis, which is critical for the growth and metastasis of solid tumors, including OC (Ferrara et al., 2003). VEGF-A promotes endothelial cell proliferation, migration, and survival through its interaction with the VEGF receptor-2 (VEGFR-2) on endothelial cells, leading to the formation of new blood vessels (Peach et al., 2018). By binding to VEGF-A, Bevacizumab obstructs this interaction, thus inhibiting the process of neovascularization (Mao et al., 2022). In addition to inhibiting angiogenesis, Bevacizumab plays a crucial role in remodeling the TME (Chen and Hurwitz, 2018). TME in tumors is often characterized by hypoxia, immune evasion, and the presence of immunosuppressive cells such as Tregs and TAMs (Sasidharan et al., 2021; Mortezaee and Majidpoor, 2021). By targeting VEGF-A, Bevacizumab not only reduces tumor blood supply but also alters the immune landscape, potentially enhancing the efficacy of concurrent immunotherapies (de Aguiar and de Moraes, 2019). Bevacizumab’s ability to normalize aberrant blood vessels may also improve the delivery of chemotherapeutic agents, allowing for better penetration and efficacy (Wong et al., 2016). The multifaceted mechanisms of Bevacizumab, ranging from anti-angiogenesis to immune modulation, underline its potential in enhancing the therapeutic response when combined with other treatment strategies, such as chemotherapy and immune checkpoint inhibitors (Lee et al., 2025; Moore et al., 2021). These dynamic processes were illustrated Figure 1, highlighting the complex interaction between VEGF-A, Bevacizumab, and the TME.

#### 2.1.1 Bevacizumab’s anti-angiogenic effect

Bevacizumab exerts its anti-angiogenic effects by specifically binding VEGF-A, preventing its interaction with VEGF receptors and thereby blocking VEGF signaling (Hsu and Wakelee, 2009). VEGF-A, the most potent member of the VEGF family, activates VEGFR-1 and VEGFR-2, which in turn trigger downstream signaling cascades such as Ras/Raf/MEK/ERK and PI3K/AKT/mTOR, promoting endothelial cell proliferation, migration, and survival (Melincovici et al., 2018; Wang et al., 2020; Iida et al., 2024). By neutralizing VEGF-A, Bevacizumab disrupts these pathways, leading to G1-phase cell cycle arrest and increased endothelial cell apoptosis, thereby halting neovascularization (Ebos and Kerbel, 2011). Bevacizumab also reduces VEGF levels, causing regression of pre-existing, structurally abnormal blood vessels. These immature vessels, lacking pericyte coverage and basement membrane integrity, collapse after VEGF inhibition, resulting in reduced vascular density and diminished tumor blood supply (Paez-Ribes et al., 2009). In addition, Bevacizumab mitigates vascular permeability and tumor-associated edema by restoring endothelial junction proteins such as VE-cadherin, which are otherwise downregulated by VEGF (Zhuang et al., 2019; Wang et al., 2006; Cianfrocca et al., 2016). It also decreases MMP activity, stabilizes the extracellular matrix (ECM), and suppresses integrin signaling, collectively reducing tumor invasiveness (van Hinsbergh and Koolwijk, 2008). The interleukin-6 (IL-6) axis also contributes to VEGF-driven angiogenesis by suppressing angiopoietin-1 (Ang-1), a process counteracted by Bevacizumab, further reinforcing its anti-angiogenic effects (Mohamed et al., 2024). Beyond these actions on endothelial cells, Bevacizumab disrupts tumor angiogenic sprouting by interfering with the VEGF/Notch pathway, which is critical for endothelial tip cell formation and vascular branching. By blocking this interaction, Bevacizumab inhibits the formation of new vascular sprouts, limiting the expansion of the tumor’s vascular network (Carmeliet and Jain, 2011; Liu ZL. et al., 2023). This multi-pronged inhibition underpins the widespread adoption of Bevacizumab in OC therapy.

#### 2.1.2 Bevacizumab remodels the TME in OC

Bevacizumab reshapes the TME by inhibiting VEGF-A, leading to a range of effects, including vascular structural repair, metabolic regulation, and the reversal of immunosuppression (Garcia et al., 2020). By blocking VEGF signaling, Bevacizumab normalizes abnormal vascular structures, as evidenced by upregulated expression of inter-endothelial junction proteins like VE-cadherin and enhanced pericyte coverage (PDGFRβ^+^), which together reduce vascular permeability and improve blood perfusion (de Aguiar and de Moraes, 2019; Darden et al., 2019). This normalization not only increases the penetration of chemotherapeutic agents, such as paclitaxel, into the tumor parenchyma but also inhibits ECM degradation by MMPs and integrin signaling, ultimately reducing tumor cell invasiveness and metastatic potential (Garcia et al., 2020; Yang T. et al., 2021; Wang K. et al., 2021).

A key aspect of Bevacizumab’s impact on the TME is the amelioration of the hypoxic environment. VEGF inhibition leads to improved tumor oxygenation, which reduces HIF-1α expression, decreases lactate accumulation, and increases pH, thereby relieving metabolic stress and reversing acidosis-induced immunosuppression (Fan et al., 2022). This enhanced oxygenation not only boosts chemotherapy efficacy but also promotes CD8^+^ T cell infiltration and cytotoxic activity while reducing the recruitment of immunosuppressive cells such as M2-type TAMs (Fanale et al., 2022; Quillien et al., 2019). Bevacizumab further reprograms the immunosuppressive TME by inducing polarization of TAMs towards a pro-inflammatory M1 phenotype (iNOS^+^, CD86^+^), decreasing the numbers of Tregs and myeloid-derived suppressor cells (MDSCs), and downregulating immune checkpoint molecule PD-L1((de Aguiar and de Moraes, 2019), (Huang et al., 2012)).

The synergy between Bevacizumab and immune checkpoint inhibitors, such as anti-PD-1/PD-L1, is particularly noteworthy (Zhang et al., 2019; Andrikopoulou et al., 2023). After vascular normalization, CD8^+^ T cells can efficiently infiltrate the tumor through the remodeled vasculature, where they release granzyme B and perforin to directly kill tumor cells (Sato et al., 2005). Additionally, enhanced dendritic cell (DC) maturation facilitates antigen presentation, further activating tumor-specific T cell responses (Fanale et al., 2022; Quillien et al., 2019). By counteracting VEGF-mediated suppression of DC function, Bevacizumab reduces Treg-mediated immunosuppression, shifting the TME from immune tolerance to immune support (Napoletano et al., 2019). Preclinical studies indicate that combining ANG-1 with Bevacizumab not only synergistically suppresses tumor stem cell properties but also suppresses M2-type TAM infiltration, further optimizing TME oxygenation and improving immune response (Sica and Mantovani, 2012).

#### 2.1.3 Comparative analysis with other Anti-VEGF agents

While bevacizumab remains the foundation of anti-angiogenic therapy in ovarian cancer, other monoclonal antibodies including ramucirumab, which targets VEGFR-2, and brolucizumab, a single-chain inhibitor of VEGF-A, also warrant consideration. Ramucirumab, approved for gastric (Fuchs et al., 2014) and lung cancers (Garon et al., 2014), demonstrates limited efficacy in ovarian cancer, primarily due to compensatory activation of alternative angiogenic pathways such as FGF signaling (Santorsola et al., 2024). Although brolucizumab’s compact molecular structure improves tissue penetration, its clinical use is restricted to ophthalmic indications due to risks of systemic toxicity (Dugel et al., 2020). In contrast, bevacizumab’s clinical superiority is reinforced by robust validation in Phase III trials such as GOG-0218 (Burger et al., 2011) and ICON7 (Perren et al., 2011), as well as its compatibility with combination therapies involving PARP inhibitors and immunotherapies.

### 2.2 Pharmacokinetics and dosing regimens

The pharmacokinetics and dosing regimens of Bevacizumab in OC treatment have been increasingly defined through clinical trials. The standard regimen, based on Phase III studies, involves intravenous administration of 15 mg/kg every 3 weeks, which has shown improvements in PFS (Danesi et al., 2021; Krasner et al., 2019). However, in platinum-resistant recurrent OC, alternative schedules such as 10 mg/kg every 2 weeks or combinations with chemotherapeutic agents like paclitaxel have been proposed to optimize the balance between efficacy and toxicity (Alqaisi et al., 2025; Soyama et al., 2020). A Phase II randomized trial highlighted that alterations in dosing schedules may impact OS, underscoring the importance of tailoring treatment to individual patient profiles (Alqaisi et al., 2025). Bevacizumab has a half-life of approximately 20 days, facilitating prolonged circulation and enhancing synergy with chemotherapy (Papachristos and Sivolapenko, 2020). It reduces tumor interstitial pressure via inhibition of the VEGF signaling pathway, improving the intratumoral penetration of paclitaxel and its therapeutic efficacy (Cesca et al., 2016). This effect is especially pronounced with nanoparticle-based formulations, such as albumin-bound paclitaxel, which further concentrates the drug in target tissues (Kalogera et al., 2024). In combination regimens, chemotherapy agents are typically administered prior to Bevacizumab to avoid interference with drug distribution (Mirza et al., 2019a). When paired with PARP inhibitors like Niraparib, Bevacizumab’s half-life remains largely unaffected; however, dose-escalation studies are warranted to assess tolerability (Lorusso et al., 2021). Optimal dosing must also account for patient-specific factors. Phase I trials demonstrate that pharmacokinetic parameters, such as the AUC of Bevacizumab, remain consistent when co-administered with other anti-angiogenic agents, like those observed with monotherapy (Hyman et al., 2018).

## 3 Clinical advances of bevacizumab in OC treatment

**FIGURE 2:**
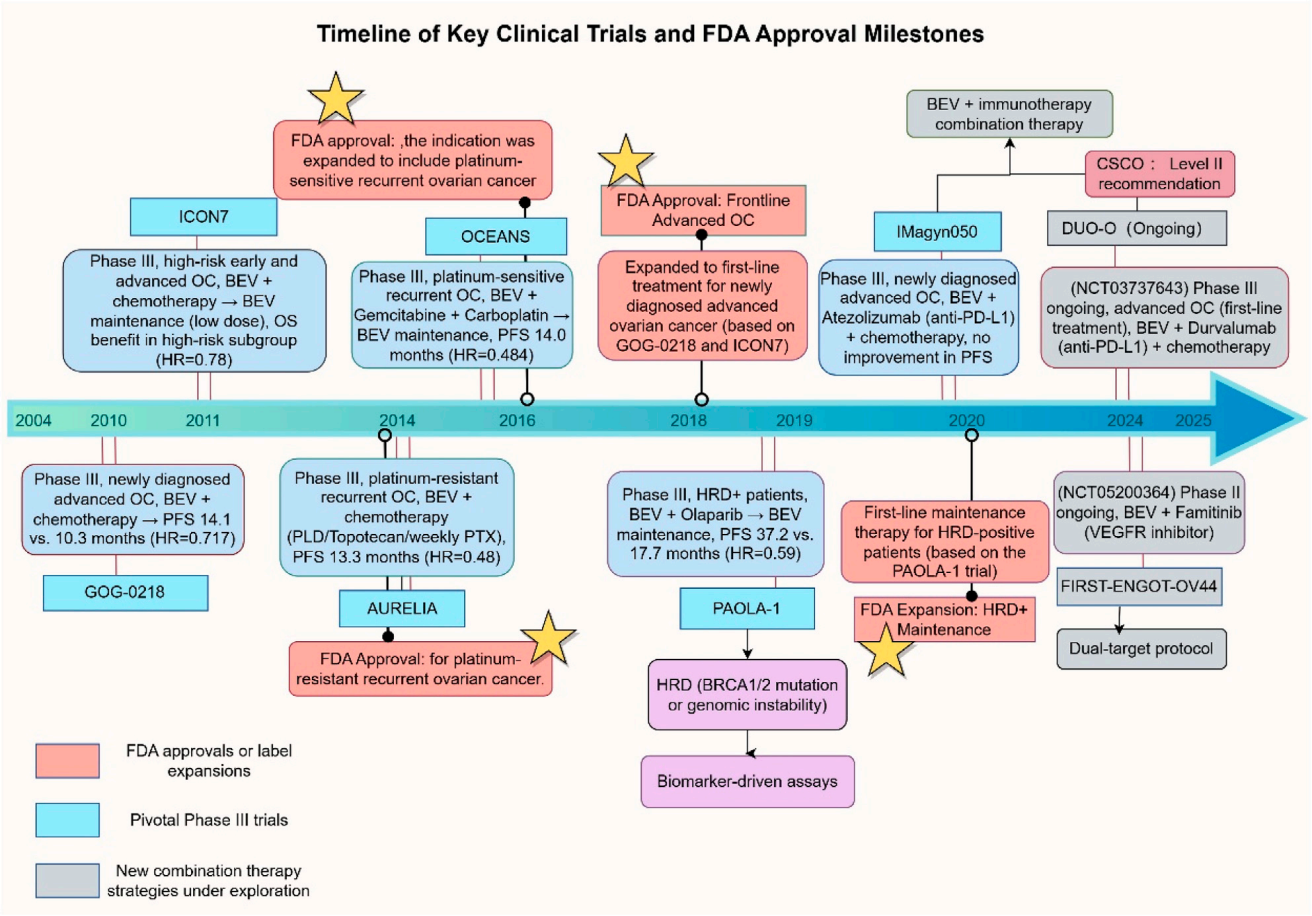
Timeline of Key Clinical Trials and FDA Approvals for Bevacizumab in OC. Abbreviations: PLD, Pegylated Liposomal Doxorubicin; PTX, Paclitaxel; HRD+, Homologous Recombination Deficiency Positive; PFS, Progression-Free Survival; OS, Overall Survival; OC, Ovarian Cancer. This figure was created by Figdraw (https://www.figdraw.com). Abbreviations: BEV, Bevacizumab; OC, ovarian cancer; HRD, homologous recombination deficiency; PFS, progression-free survival; OS, overall survival; HR, hazard ratio; PLD, pegylated liposomal Doxorubicin. PXT, Paclitaxel. Data sources: *ClinicalTrials.gov*, FDA labels, and published trial results.

Bevacizumab, the first monoclonal antibody targeting VEGF, revolutionized anti-angiogenic therapy for solid tumors following its approval in 2004 for metastatic colorectal cancer (Hurwitz et al., 2004) (Figure 2). Its clinical application in OC began with early exploratory studies by Monk and colleagues, who reported a 21% ORR with single-agent therapy in advanced refractory OC, laying the groundwork for subsequent Phase III trials (Monk et al., 2009). Over the past decade, Bevacizumab’s clinical adoption has been bolstered by an expanding body of evidence, with several pivotal Phase III studies solidifying its role in OC management, including key indications such as first-line treatment, platinum-sensitive/resistant recurrence, and maintenance therapy (Table 1).

**TABLE 1:** Summary of Bevacizumab-Based Regimens in OC.

Acronym	Patient Cohort	Regimen	Control	Primary Outcomes	Notes	References
GOG-0218	Newly diagnosed advanced OC (FIGO III/IV) post-debulking	BEV 15 mg/kg ^+^ Carboplatin/PTX → BEV maintenance	Placebo + Carboplatin/PXT → placebo maintenance	PFS: 14.1 vs. 10.3 months; OS: No significant difference	First trial establishing BEV in frontline therapy	Liu et al. (2025a)
ICON7	High-risk early-stage or advanced OC	BEV 7.5 mg/kg + Carboplatin/PTX → BEV maintenance	Chemotherapy alone	PFS: 22.4 vs. 24.1 months; OS: Subgroup in high-risk patients 28.8vs36.6 months	Lower BEV dose used; OS benefit in high-risk subgroup	Soyama et al. (2020)
OCEANS	Platinum-sensitive recurrent OC	BEV 15 mg/kg q3w + Gemcitabine/ Carboplatin→ BEV maintenance	Placebo + Gemcitabine/Carboplatin → placebo maintenance.	median PFS: 12.4 vs. 8.4 months	First trial in platinum-sensitive recurrence	Burger et al. (2011)
AURELIA	Platinum-resistant recurrent OC	BEV 15 mg/kg + PLD/Topotecan/weekly PTX	Chemotherapy alone	PFS: 6.7 vs. 3.4 months	BEV + chemotherapy as salvage therapy	Aghajanian et al. (2012)
PAOLA-1	Advanced OC with HRD^+^	BEV 15 mg/kg + Olaparib maintenance. after chemo + BEV	BEV + placebo maintenance	PFS: 37.2 vs. 17.7 months in HRD^+^ subgroup	Biomarker-driven strategy	Papachristos and Sivolapenko (2020)
IMagyn050	Newly diagnosed advanced OC	BEV 15 mg/kg + Atezolizumab + Carboplatin/PTX → BEV + Atezolizumab maintenance	BEV + Placebo + chemotherapy → BEV + Placebo maintenance	PD-L1-positive population PFS: 20.8 vs. 18.5 months	First BEV + immunotherapy combination trial	Mortezaee and Majidpoor (2021), Cesca et al. (2016)
ATALANTE	Advanced OC (ongoing,)	Carboplatin/PXT + BEV 15 mg/kg + Atezolizumab → BEV + Atezolizumab maintenance	BEV + chemotherapy → BEV + Placebo maintenance	Primary endpoint: PFS (Researchers evaluate)	Triple-combination strategy	Park et al. (2024)
DUO-O	Advanced OC (ongoing,)	BEV + Durvalumab + Carboplatin/PXT → BEV + Durvalumab + Olaparib maintenance	BEV + chemotherapy → BEV + Placebo maintenance	Primary endpoint: PFS (results pending)	Triple-combination strategy	NCT03737643.

Abbreviations: BEV, Bevacizumab; OC, ovarian cancer; HRD, homologous recombination deficiency; PFS, progression-free survival; OS, overall survival; HR, hazard ratio; PLD, pegylated liposomal Doxorubicin. PXT, Paclitaxel. Data sources: ClinicalTrials.gov, FDA labels, and published trial results.

### 3.1 Bevacizumab in first-line therapy for advanced OC

The role of Bevacizumab in first-line treatment for advanced OC was solidified through two pivotal Phase III trials, GOG-0218 and ICON7. The GOG-0218 study (NCT00262847) demonstrated that Bevacizumab, when combined with carboplatin-paclitaxel chemotherapy and followed by maintenance therapy, significantly prolonged median PFS, but it did not significantly affect OS (Burger et al., 2011). A trend toward improved overall survival (OS) was observed in the stage IV subgroup, suggesting that tumor biological characteristics may influence Bevacizumab’s efficacy (Tewari et al., 2019). The ICON7 study (NCT00483782), which administered a lower dose of Bevacizumab in combination with chemotherapy, confirmed a significant OS benefit in the high-risk subgroup. In this group, the OS was extended to 39.3 months in the Bevacizumab arm, compared to 34.5 months in the control arm (Gonzalez Martin et al., 2019). This underscores the importance of patient stratification in predicting treatment outcomes. In unresectable advanced OC, the ANTHALYA studies evaluated Bevacizumab in combination with neoadjuvant chemotherapy, reporting an increased rate of pathological complete resection (Rouzier et al., 2017; Li Y. et al., 2017). However, no significant improvement was observed in the rate of complete cytoreduction (R0), and Phase III evidence remains lacking. The MITO16A study further revealed that while neoadjuvant chemotherapy combined with Bevacizumab reduced tumor volume, it did not lead to a significant increase in surgical success rates, suggesting that Bevacizumab’s vascular normalization effects may be constrained by TME heterogeneity (Daniele et al., 2017). Nevertheless, MITO16A confirmed that the combination of Bevacizumab and chemotherapy significantly prolonged both PFS and OS in high-risk patients with OC, such as those with stage IIIC/IV disease or postoperative residual lesions >1 cm (Daniele et al., 2017). Moreover, interval debulking surgery (IDS) following Bevacizumab and chemotherapy did not significantly increase perioperative complications, such as wound infection or intestinal obstruction, supporting its use in high-risk patients (Daniele et al., 2017). In contrast to the ICON7 trial, MITO16A utilized the same 15 mg/kg dose as the GOG-0218 study, while ICON7 employed 7.5 mg/kg, indicating that dose intensity may influence efficacy. The recent GOG-0218 trial confirmed that maintenance Bevacizumab therapy in ICON-7 was effective for patients exhibiting poor chemotherapy sensitivity (You et al., 2022). Takamatsu et al. further demonstrated that, in first-line treatment, Bevacizumab may offer greater benefit to patients with a poorer prognosis and a lower likelihood of disease rebound (Takamatsu et al., 2023). Therefore, Bevacizumab maintenance therapy should be prioritized for high-risk patients and those with poor chemotherapy sensitivity, with treatment decisions based on the patient’s KELIM score, considering both dose intensity and safety.

### 3.2 Bevacizumab in recurrent OC

The efficacy of Bevacizumab in recurrent OC has been established in two pivotal trials: OCEANS and AURELIA. The OCEANS study (NCT00434642) in platinum-sensitive recurrent patients revealed that Bevacizumab combined with gemcitabine-carboplatin chemotherapy followed by maintenance therapy significantly improved median PFS and ORR (Aghajanian et al., 2012). These findings support Bevacizumab as a standard treatment for platinum-sensitive recurrent patients, though further investigation into its long-term benefits is warranted. In platinum-resistant recurrent patients, the AURELIA study (NCT00976911) demonstrated that bevacizumab combined with non-platinum chemotherapy significantly enhanced median PFS, particularly benefiting patients with ascites, likely through the regulation of vascular permeability to mitigate intraperitoneal fluid accumulation (Pujade-Lauraine et al., 2014). In biomarker-driven therapy, the combination of bevacizumab with PARP inhibitors has shown promising potential. The PAOLA-1 study (NCT02477644) revealed that Bevacizumab plus olaparib maintenance therapy significantly extended median PFS to 37.2 months in patients with homologous recombination deficiency (HRD), with the BRCA-mutated subgroup achieving an unmeasurable PFS. However, in patients with wild-type BRCA, the improvement in OS was limited, suggesting that HRD may serve as a more reliable predictive biomarker (Ray-Coquard et al., 2019). The AVANOVA study further investigated the efficacy of Niraparib in combination with Bevacizumab, demonstrating a significant prolongation of PFS in patients with platinum-sensitive recurrent OC. However, this was accompanied by an increased incidence of grade 3 or higher hypertension, necessitating a careful balance between therapeutic benefit and toxicity (Mirza et al., 2019a). The synergistic mechanism between Bevacizumab and PARP inhibitors may involve vascular normalization, which enhances drug delivery, though the indirect modulation of DNA damage repair pathways requires further investigation (Lorusso et al., 2024). The role of hyperthermic intraperitoneal chemotherapy (HIPEC) in recurrent OC remains uncertain, despite improvements in OS when applied during interval cytoreductive surgery. The CHIPOR study examining HIPEC combined with Bevacizumab showed that postoperative HIPEC extended PFS but did not improve OS, while increasing toxicity, underscoring the need for a comprehensive risk-benefit assessment (Classe et al., 2024).

### 3.3 Bevacizumab in maintenance therapy

The PAOLA-1/ENGOT-ov25 study significantly expanded the role of Bevacizumab in maintenance therapy for OC. This phase III trial evaluated Bevacizumab plus Olaparib as first-line maintenance therapy for newly diagnosed advanced OC, yielding a substantial prolongation of PFS in the HRD^+^ subgroup, particularly in BRCA-mutated patients, with median PFS not reached (Ray-Coquard et al., 2019). In HRD^+^ patients with wild-type BRCA, the median PFS was 28.3 months, indicating that HRD status may serve as an independent predictive marker, although its optimal threshold requires further validation (Lorusso et al., 2024). Long-term follow-up data revealed a 5-year survival rate of 65.5% in the combination therapy group, compared to 48.2% in the control group. However, the OS difference across the entire study population did not achieve statistical significance, highlighting the need for precise patient stratification to optimize therapeutic efficacy (Lorusso et al., 2024). The PAOLA-1 studies confirmed that patients with FIGO stage III/IV disease, postoperative residual lesions <1 cm, and HRD^+^ status experienced the greatest benefit, with a median PFS of 46.8 months, while HRD-negative patients, regardless of BRCA status, showed no significant PFS improvement (Ray-Coquard et al., 2019; Lorusso et al., 2024). These findings advocate for the integration of HRD testing into routine molecular profiling before initiating maintenance therapy, though clinical implementation must consider the balance between testing costs and accessibility. Additionally, the increased toxicity observed with Bevacizumab combined with PARP inhibitors warrants attention. In the PAOLA-1 study, 19% of patients receiving combination therapy developed grade 3 or higher hypertension, compared to 5% in the Bevacizumab monotherapy group, while hematologic toxicities such as anemia and neutropenia increased by 12%–15% compared to Olaparib monotherapy. This underscores the need for dose adjustments and continuous monitoring to optimize tolerability (Ray-Coquard et al., 2019; Lorusso et al., 2024). In patients previously treated with Bevacizumab, the AVANOVA2/ENGOT-ov24 study demonstrated that Niraparib combined with Bevacizumab further extended median PFS in platinum-sensitive recurrent maintenance therapy. However, this combination was associated with a significantly higher risk of intestinal perforation and thrombocytopenia, highlighting the need for careful evaluation of vascular integrity and bone marrow function in multi-line therapy patients (Mirza et al., 2019b). Future research should focus on optimizing the sequencing of combination therapies, such as intermittent dosing, and exploring novel biomarkers, like angiogenesis-related gene expression profiles, to achieve a balance between efficacy and safety.

### 3.4 Bevacizumab in novel combination strategies

Bevacizumab, a cornerstone of anti-angiogenic therapy, is catalyzing a paradigm shift in OC treatment through innovative combination strategies, moving from empirical regimens to precision approaches driven by underlying mechanisms. Recent advancements in multidimensional synergistic therapies, incorporating immune modulation, DNA repair interventions, multi-target pathway inhibition, and biomarker stratification, aim to overcome drug resistance and extend survival. However, variability in therapeutic efficacy, including inconsistent OS benefits and differing biomarker responses, underscores the urgent need for precise patient stratification. To address the survival challenges in OC, several ongoing trials, such as NCT05116189 and NCT04953879, are exploring novel multi-drug combinations, as outlined in Table 2.

**TABLE 2:** Ongoing clinical trials exploring novel bevacizumab combinations in OC.

Trial Acronym	Patient Population	Intervention	Primary Endpoint	Key Innovations	ClinicalTrials.gov ID
DUO-O	Newly diagnosed advanced OC (HRD^+^)	Arm A: BEV + Durvalumab + chemo → BEV + Durvalumab + Olaparib maintenanceArm B: BEV + chemo → BEV + placebo maintenance	PFS (HRD^+^ subgroup)	Triple therapy (BEV + PD-L1 inhibitor + PARPi)	NCT03737643
ATALANTE	Platinum-sensitive recurrent OC	Arm 1: BEV + Niraparib Arm 2: BEV + Mirvetuximab Soravtansine (ADC) Arm 3: BEV + standard chemo	PFS (comparative efficacy)	Novel ADC and PARPi combinations	NCT02891824
BEACON	Platinum-resistant OC with high VEGF expression	BEV + Cabozantinib (VEGFR/MET inhibitor)	ORR	Dual anti-angiogenic targeting	NCT04729622
MIMOSA	Recurrent OC with ascites	Arm A: BEV + Pembrolizumab + chemo Arm B: BEV + chemo	Ascites reduction at 12 weeks	Immuno-angiogenic synergy in ascites control	NCT05200364
GARNET	Advanced OC with FGFR alterations	BEV + Erdafitinib (FGFR inhibitor)	MTD, ORR	Targeting FGFR pathway resistance	NCT04919642
OVARIO	Advanced OC with BRCA wild type	BEV + Adavosertib (WEE1 inhibitor) + Olaparib	6-month PFS rate	Synthetic lethality with DNA damage repair inhibitors	NCT03579316
ATHENA	Newly diagnosed OC	Arm A: BEV + Rucaparib (HRD^+^ subgroup) Arm B: BEV + chemo (HRD^−^ subgroup)	PFS (biomarker-driven)	Biomarker-guided adaptive design	NCT03522246
GLORIOSA	Fra – high PSOC	BEV + MIRV	PFS :13.3 months	MIRV plus Bevacizumab	NCT05445778

Abbreviations: BEV, bevacizumab; OC, ovarian cancer; HRD, homologous recombination deficiency; PFS, progression-free survival; ORR, objective response rate; ADC, antibody-drug conjugate; PARPi, PARP, inhibitor; MTD, maximum tolerated dose; PSOC, platinum-sensitive ovarian cancer; MIRV, Mirvetuximab Soravtansine - gynx. Data sources: ClinicalTrials.gov and References.

#### 3.4.1 PD-1/PD-L1

In the realm of immune combination therapies targeting microenvironment remodeling and therapeutic efficacy heterogeneity, Bevacizumab enhances the T-cell cytotoxic effects of PD-1/PD-L1 inhibitors by mitigating the VEGF-driven immunosuppressive microenvironment, which includes reducing the infiltration of regulatory T cells and MDSCs (Jain, 2005). The Phase III DUO-O study (NCT03737643) assessed a triple combination of Bevacizumab, durvalumab (anti-PD-L1), and Olaparib, achieving a 5.8-month prolongation of PFS in HRD^+^ newly diagnosed patients (Harter et al., 2019). However, OS did not reach statistical significance, indicating the need for optimized timing of immune activation and refined patient selection (Harter et al., 2019). Rosario et al. conducted a Phase II trial (NCT02853318) evaluating a triple therapy comprising the anti-PD-1 drug pembrolizumab, the anti-VEGF drug Bevacizumab, and oral Cyclophosphamide (Rosario et al., 2024). This combination significantly extended the median PFS of patients with recurrent OC to 10.2 months, with a disease control rate of 95%, providing promising treatment for platinum-resistant patients. Moreover, increased levels of glutamate, serine, and trimethylamine-N-oxide (TMAO) in patients’ feces were linked to enhanced CD8^+^ T cell function, suggesting that this triple therapy modulates the tumor immune microenvironment by altering the gut microbiota composition and metabolite levels (Rosario et al., 2024). The IMagyn050 study (NCT03038100) revealed that Bevacizumab plus atezolizumab benefits only PD-L1-positive patients in terms of PFS, highlighting the importance of TME heterogeneity. However, immune-related adverse events, including colitis and pneumonia, as well as proteinuria and hypertension associated with Bevacizumab, necessitate dynamic monitoring (Krishnan et al., 2023).

#### 3.4.2 PARP inhibitors

Bevacizumab enhances the intratumoral delivery efficiency of PARP inhibitors by inducing tumor vascular normalization, while concurrently targeting angiogenesis and DNA repair pathways for synergistic effects (Fukumura et al., 2018; Zhang C. et al., 2024; Dai et al., 2024). The ATALANTE/ENGOT-ov29 phase III clinical trial demonstrated that in patients with recurrent OC and a progression-free interval (PFI) > 6 months, a triple therapy combining atezolizumab, Bevacizumab, and chemotherapy increased median overall survival (OS) to 35.5 months in the PD-L1-positive subgroup. However, the concomitant rise in immune-related adverse events underscores the need for further investigation into the OC immune microenvironment to optimize treatment strategies (Kurtz et al., 2023). The OVARIO Phase II trial revealed that the combination of niraparib and Bevacizumab significantly extended PFS to 28.3 months in patients with HRD without a significant increase in toxicity, suggesting greater potential in biomarker-selected patients. Nonetheless, hematologic toxicity remains a concern (Hardesty et al., 2022). Challenges persist, as HRD-negative patients exhibit limited responses to PARP inhibitors plus Bevacizumab, and genomic instability may accelerate clonal evolution. The ATHENA study (NCT03522246) employed an HRD-stratified approach (Bevacizumab plus Rucaparib or chemotherapy), highlighting the need for careful attention to cumulative toxicities during maintenance therapy (Monk et al., 2022). To address the gap in effective maintenance treatment for BRCA wild-type platinum-sensitive recurrent OC, Lee et al. conducted the phase II OPEB-01 trial (NCT04361370), which investigates the synergistic effects of Olaparib (PARP inhibitor), Pembrolizumab (PD-1 inhibitor), and Bevacizumab (anti-angiogenic agent), exploring potential treatment regimens for this patient subgroup (Lee et al., 2021).

#### 3.4.3 Additional therapeutic approaches

Multi-target inhibitors combined with Bevacizumab aim to disrupt compensatory angiogenic escape mechanisms and overcome resistance, particularly targeting the compensatory activation of the VEGF pathway, including HGF/MET and FGF/FGFR signaling. The Phase II BEACON study (NCT03363867) assessed Bevacizumab plus Cabozantinib, a VEGFR/MET inhibitor, in VEGF-high, platinum-resistant patients, achieving an ORR of 32%. However, the increased risks of hypertension and hepatotoxicity warrant cautious implementation (Manoharan et al., 2024). Heublein et al. conducted an exploratory analysis based on the AGO-OVAR11/ICON-7 phase III trial, suggesting that combining FGFR inhibitors with Bevacizumab could enhance anti-angiogenic efficacy by targeting the FGFR/FGF signaling pathway, such as inhibiting FGFR1/FGFR4, and leveraging the protective effects of FGF19. This combination could overcome Bevacizumab resistance and extend survival, providing a theoretical foundation for biomarker-driven precision combination therapy in patients with OC exhibiting high FGFR expression (Heublein et al., 2024). Dynamic adjustment strategies informed by molecular subtyping are reshaping treatment decisions. The combination of MIRV and Bevacizumab has shown promising results in patients with platinum-sensitive OC (PSOC) and platinum-resistant OC (PROC) across all levels of FRα expression (FORWARD II Phase trial, NCT02606305) (O'Malley et al., 2021; O'Malley et al., 2019). These trials indicate that the MIRV-Bevacizumab combination demonstrates an encouraging ORR and a favorable tolerability profile in patients with recurrent OC, particularly those with tumors exhibiting high FRα expression. However, Phase III validation is still needed (O'Malley et al., 2021; O'Malley et al., 2019). Recently, O'Malley et al. conducted the Phase III GLORIOSA trial (NCT05445778) in patients with FRα-high PSOC. This trial evaluates MIRV combined with Bevacizumab as a novel maintenance regimen for patients whose disease has not progressed after second-line platinum-based chemotherapy plus Bevacizumab, aiming to identify better long-term treatment options (O'Malley et al., 2024). The DUO-O study stratified patients by HRD status, limiting triple maintenance therapy to HRD^+^ individuals to prevent overtreatment, while the ATHENA trial used an adaptive design for real-time allocation of Bevacizumab plus PARP inhibitors or chemotherapy (Harter et al., 2019). However, inconsistencies in HRD detection thresholds and PD-L1 scoring criteria hinder cross-study comparability. Furthermore, clonal evolution under therapeutic pressure, including dynamic variations in circulating tumor DNA (ctDNA), may compromise the predictive value of biomarkers, highlighting the need for real-time monitoring through advanced techniques such as single cell sequencing and spatial transcriptomics (Paspalj et al., 2024). Nelson et al. confirmed the feasibility of combining Bevacizumab with Temsirolimus (an mTOR inhibitor) and Valproic acid (an HDAC inhibitor) for advanced cancer treatment in a Phase I clinical trial (NCT01552434), identifying the maximum tolerated dose. However, this combination regimen exhibited significant concomitant toxicity, with an ORR of only 7.9%, suggesting that strict management of toxicity is essential to support its future development (Nelson et al., 2023). Organoid models and AI-driven toxicity prediction tools may expedite the screening of combination regimens, while optimizing endpoints should integrate patient-reported outcomes alongside radiomic biomarkers, such as perfusion MRI, to assess vascular normalization (Scalia et al., 2025; Ambreen et al., 2025).

## 4 Challenges and limitations

### 4.1 Toxicity and safety concerns

The widespread use of Bevacizumab in OC treatment has significantly improved patient survival, but its safety remains a critical concern in clinical practice (Coleman et al., 2017). Hypertension is one of the most common adverse effects associated with Bevacizumab, with incidence rates ranging from 18% to 35% across multiple studies. While typically manageable with antihypertensive medications, severe hypertension in certain patients may necessitate dose adjustments or discontinuation of treatment (Wang Y. et al., 2023). Proteinuria, occurring in approximately 21%–30% of patients, is another frequent toxicity, generally mild to moderate in severity. However, severe proteinuria (≥ Grade 3) can result in renal impairment, necessitating close monitoring (Wu et al., 2024). Gastrointestinal perforation, although rare, is a potentially fatal complication, with an incidence of 1%–2%, particularly in patients with a history of multiple abdominal surgeries or intestinal involvement (Coleman et al., 2017). Furthermore, the risk of wound healing complications is significantly elevated in patients receiving Bevacizumab shortly after surgery, and clinical guidelines recommend a minimum treatment-free interval of 4–6 weeks post-surgery to mitigate these risks (Kobayashi et al., 2020). Risk-benefit assessments should be individualized, especially for specific populations. Elderly patients (≥70 years) tend to have a reduced tolerance to Bevacizumab, with studies reporting a higher incidence of hypertension and thrombotic events compared to younger cohorts (Wang Y. et al., 2023). However, survival benefits in this group appear comparable to those in younger patients, emphasizing the need for careful patient selection and enhanced toxicity monitoring (Selle et al., 2018). Patients with comorbid cardiovascular disease or chronic kidney disease are at higher risk of hypertension and proteinuria, necessitating careful consideration of the survival benefits relative to the potential quality-of-life decrements due to exacerbated toxicity (Kose et al., 2020). Notably, a real-world study suggests that with stringent dose management and toxicity monitoring, Bevacizumab can maintain a manageable safety profile even in patients with comorbidities (Zhang et al., 2023). In conclusion, the toxicity profile of Bevacizumab necessitates thorough baseline patient evaluation before treatment, dynamic monitoring of adverse reactions during therapy, and a cautious balance of efficacy and safety in specific patient populations (Chitoran et al., 2024). Future research should focus on identifying biomarkers that can accurately delineate high-risk groups, optimizing individualized treatment strategies.

### 4.2 Bevacizumab resistance

**FIGURE 3:**
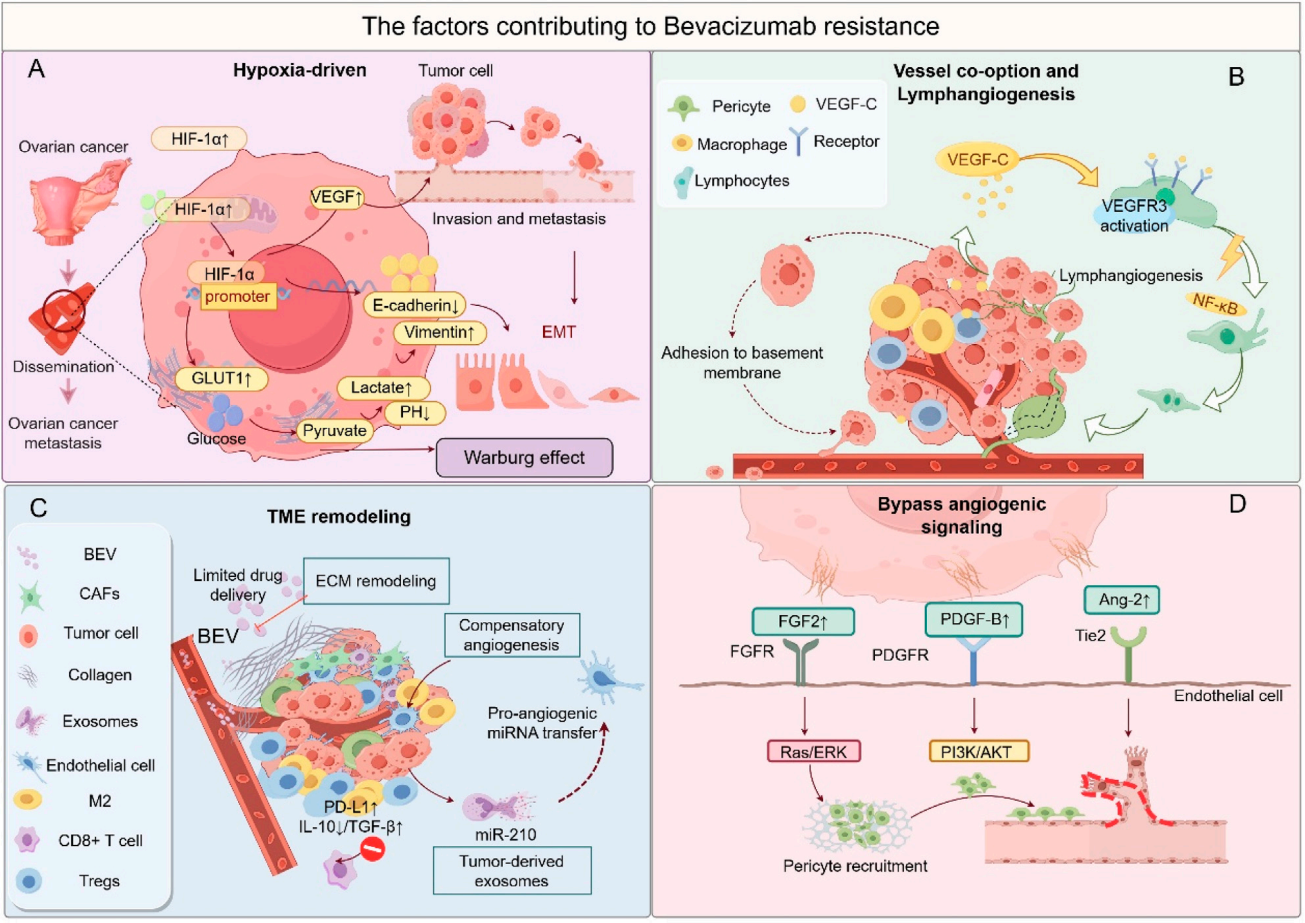
The factors contributing to bevacizumab resistance. **(A)** Hypoxia-driven malignancy: HIF-1α-mediated EMT, metabolic reprogramming, and metastasis. **(B)** Vessel co-option and lymphangiogenesis: Tumor cells exploit pre-existing vessels or induce lymphatic expansion. **(C)** Immunosuppressive TME: Infiltration of M2 macrophages and Tregs, along with stromal fibrosis. **(D)** Bypass angiogenesis: Activation of alternative pro-angiogenic pathways (FGF/FGFR, PDGF/PDGFR, Ang-2/Tie2). This figure was created by Figdraw (https://www.figdraw.com).

Resistance to Bevacizumab poses a significant challenge in limiting its long-term efficacy, driven by complex dynamic interactions among tumor cells, the vascular system, and the immune TME (Figure 3). The mechanisms underlying this resistance can be classified into four major categories, with notable synergistic effects between these pathways.

The activation of alternative angiogenic pathways is the primary mechanism of Bevacizumab resistance. When VEGF/VEGFR signaling is inhibited, tumors compensate by upregulating alternative pathways, such as FGF/FGFR and Angiopoietin-2/Tie2, to maintain angiogenesis. For instance, in the AURELIA study involving platinum-resistant patients, tumor tissue demonstrated a 3.2-fold increase in FGF2 mRNA expression, while the combination of FGFR inhibitors raised the ORR to 27% (Heublein et al., 2024). Preclinical models have shown that dual targeting of VEGF and Ang-2 reduced vascular density by 58% (Zitzmann-Kolbe et al., 2023). These alternative pathways create a positive feedback loop with an immunosuppressive microenvironment: PDGF activates CAFs to secrete IL-6, promoting Treg expansion, which, in turn, enhances PDGF release, perpetuating a pro-angiogenic and immunosuppressive cycle (Horikawa et al., 2020). Vascular co-option and structural remodeling are additional critical mechanisms of resistance. Tumor cells bypass Bevacizumab’s anti-angiogenic effects by co-opting mature host vasculature or inducing lymphangiogenesis. In a Bevacizumab-resistant OC liver metastasis model, co-opted vessels constituted 42% of the vasculature, strongly correlating with elevated expression of hypoxia-inducible factor HIF-2α (Boso et al., 2023). Concurrently, post-Bevacizumab treatment, upregulation of VEGF-C/D-VEGFR3 signaling led to a 35% increase in lymphatic vessel density, facilitating tumor metastasis via the lymphatic system (Li et al., 2019). This process is closely linked to the hypoxic microenvironment: reduced blood flow from vessel co-option exacerbates hypoxia, which upregulates VEGF-C through HIF-1α, forming a self-reinforcing “hypoxia-lymphangiogenesis-metastasis” loop (Yue and Lu, 2023). Hypoxia-driven malignant transformation further amplifies resistance. Bevacizumab-induced vascular pruning triggers hypoxia, which activates HIF-mediated metabolic reprogramming and EMT. Single-cell sequencing revealed a 2.5-fold upregulation of glycolytic genes (HK2, LDHA) in resistant tumors, with increased lactate secretion promoting M2 polarization of TAMs (Lin et al., 2024). Hypoxia also elevates ZEB1/2 activity, increasing the proportion of CD44^+^CD117^+^ cancer stem cells from 5% to 18%, thereby enhancing tumor invasiveness (Nunes et al., 2024). Hypoxia-related factors, such as PGE2, also recruit MDSCs via the CXCL12/CXCR4 axis, suppressing CD8^+^ T-cell function and establishing a synergistic hypoxia-immunosuppression resistance mechanism (Abiko et al., 2021). Reprogramming of the TME sustains resistance progression. Although Bevacizumab may transiently enhance antitumor immunity in the early stages, prolonged therapy shifts the TME toward immunosuppression: the proportion of M2-type TAMs increases from 25% to 48%, inhibiting T-cell activity through IL-10 and TGF-β secretion (Boso et al., 2023); peripheral blood Treg levels rise 1.8-fold, and tumor cell PD-L1 expression significantly increases (Konstantinopoulos et al., 2019). This immunosuppression interacts with alternative angiogenic pathways, promoting M2 polarization via STAT3 signaling, while M2 macrophages secrete FGF2, creating a feedback loop that enhances resistance (Heublein et al., 2024). Additionally, prolonged anti-VEGF therapy may induce epigenetic dysregulation, such as the downregulation of miR-143-3p, leading to excessive PAI-1 secretion, which disrupts vascular structure and accelerates resistance (Yagi et al., 2023).

### 4.3 Limitations of biomarkers

The use of Bevacizumab in OC treatment faces substantial challenges, primarily due to the insufficient validation of biomarkers. Despite efforts to predict Bevacizumab efficacy using circulating VEGF levels or angiogenesis-related gene signatures, such as VEGF receptor mutations and HRD status, the clinical validation of these biomarkers exhibits considerable heterogeneity (Califano et al., 2021; Gao et al., 2021). For example, the MITO16A/MaNGO-OV2 trial revealed significant variability in the correlation between angiogenesis-related gene expressions, including VEGFA and ANGPT2, and response to Bevacizumab across different patient subgroups, with statistical significance absent in some cohorts (Califano et al., 2021). This limitation may be attributed to the dynamic activation of alternative pathways, including FGF/PDGF signaling, and the reduced predictive power of single biomarkers due to intratumoral heterogeneity (Jin et al., 2022; Ma et al., 2021). Additionally, studies investigating plasma VEGF concentration as a potential biomarker have yielded conflicting results. Some retrospective analyses report no significant association with PFS, suggesting that relying solely on VEGF levels is insufficient for guiding clinical decisions (Gao et al., 2021; Buechel et al., 2021). The challenge of patient stratification and personalized therapy is further compounded by the dynamic and complex nature of biomarkers. While genomic technologies have enabled molecular subtyping, the high genomic instability of OC makes single biomarkers inadequate for comprehensively reflecting treatment responses (You et al., 2022; Li et al., 2021). For instance, in the PAOLA-1 trial, Bevacizumab combined with Olaparib improved survival in certain patients based on HRD status; however, around 30% of HRD^+^ patients did not benefit, highlighting the limitations of current stratification strategies (Harter et al., 2022). Moreover, treatment-induced clonal evolution may further undermine the predictive value of biomarkers. Bevacizumab therapy can induce resistance via CD63-mediated VEGF sequestration in the TME, a dynamic alteration that baseline assessments struggle to capture (Ma et al., 2021; Li et al., 2021). Current research is predominantly limited to retrospective analyses or small-scale prospective studies, lacking multicenter, large-sample validation, which hampers the clinical implementation of biomarkers (Califano et al., 2021; Gao et al., 2021). For example, a retrospective study on Bevacizumab combined with chemotherapy proposed that lactate dehydrogenase (LDH) levels and the KELIM score could serve as predictive tools, but validation across independent cohorts remains inconsistent (You et al., 2022; Liu et al., 2025b). To date, no biomarker has been identified to define a patient subgroup responsive to Bevacizumab. Based on the MITO16A/MaNGO OV-2 trial, Bignotti et al. found, through next-generation sequencing for TP53 mutation assessment and immunohistochemistry for p53 expression evaluation, that unclassified missense TP53 mutations identify a patient subgroup with significant survival advantages. This mutation profile may serve as a potential prognostic marker for patients with OC receiving upfront Bevacizumab combined with chemotherapy (Bignotti et al., 2025). Moving forward, the integration of multi-omics data, including metabolomics and spatial transcriptomics, along with dynamic monitoring strategies, will be crucial for developing more comprehensive predictive models and overcoming existing limitations (Nunes et al., 2024; Liu X. et al., 2021).

### 4.4 Cost-effectiveness and accessibility

The application of Bevacizumab in OC treatment is significantly hindered by inadequate biomarker validation and limitations in stratification strategies. While biomarkers such as VEGF levels, genetic polymorphisms, and tumor mutational burden have been extensively explored, their predictive efficacy remains limited due to the absence of standardized validation criteria (Lu et al., 2024).

For instance, retrospective analyses have revealed significant heterogeneity in the relationship between plasma VEGF-A levels and Bevacizumab efficacy across studies. Consequently, using tumor VEGF-A expression as a surrogate marker for predicting response to VEGF inhibitor therapy in OC requires caution (Soyama et al., 2020). Genetic factors, such as VEGF receptor polymorphisms, have been linked to survival benefits in specific cohorts; however, validation data from Asian populations remains inadequate, restricting their broader clinical applicability (Zhao et al., 2020). The complexity of patient stratification and personalized treatment further exacerbates cost-effective challenges. Although existing stratification models, such as those combining HRD status with VEGF-D levels, enhance predictive accuracy, they rely on costly multi-omics profiling, making them impractical for widespread use in resource-limited settings (Rognoni et al., 2024). Additionally, the spatiotemporal heterogeneity of the TME complicates biomarker assessment, as single-time-point measurements may not accurately reflect treatment responses. For example, ctDNA exhibits a false-negative rate of up to 40% in patients with peritoneal metastases, while the feasibility of repeat biopsies is limited by technical barriers and patient compliance (Werner et al., 2021). The economic burden and disparities in accessibility are particularly pronounced. While biosimilars such as BEVZ92 can reduce treatment costs by up to 70%, their availability in low- and middle-income countries remains under 30%, with cold-chain logistics further restricting their use in remote areas (Yang J. et al., 2021). Adaptive clinical trial designs, such as the STAMP trial, present an opportunity to optimize resource allocation through dynamic treatment adjustments. However, their successful implementation depends on robust molecular diagnostic infrastructure and effective interdisciplinary collaboration networks (Ma et al., 2024). Moving forward, the development of low-cost, rapid diagnostic technologies, such as microfluidic chips, and the validation of stratification models’ cost-effectiveness through global data-sharing platforms are crucial to achieving equitable precision medicine.

## 5 Future perspectives

**FIGURE 4:**
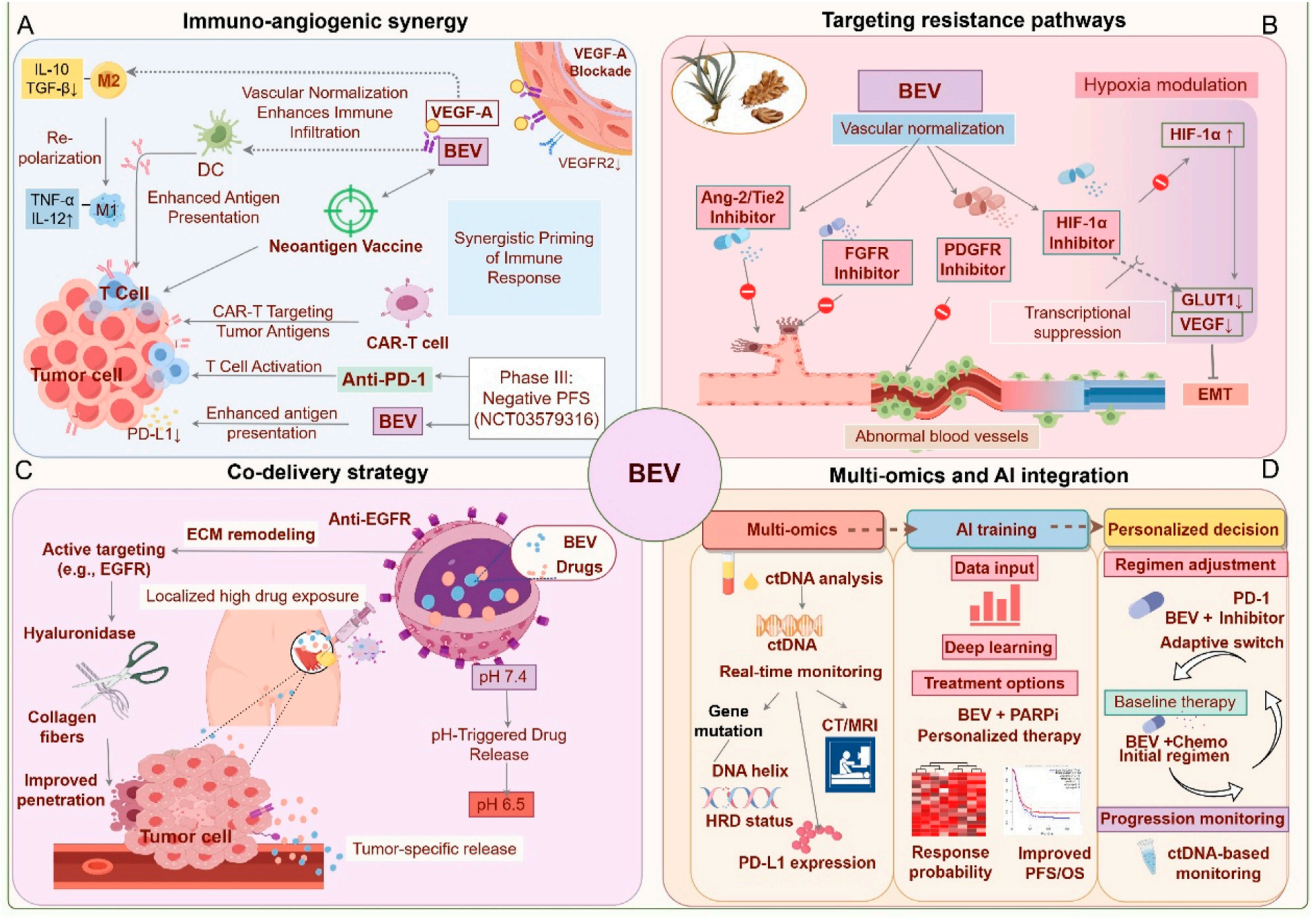
Future application strategies for Bevacizumab. **(A)** Immuno-angiogenic synergy (Blue): Bevacizumab synergizes with PD-1 inhibitors and vaccines to activate T cells and reprogram macrophages. **(B)** Targeting resistance pathways (Red): Dual inhibition of angiogenesis (Ang-2/FGFR) and hypoxia signaling to overcome adaptive resistance. **(C)** Innovative drug delivery systems (Purple): Nanoparticles and localized delivery enhance tumor targeting and reduce toxicity. **(D)** Biomarker-driven precision medicine (Orange): Dynamic biomarker monitoring and machine learning guide personalized therapy. This figure was created by Figdraw (https://www.figdraw.com).

Bevacizumab is positioned to drive multidimensional, synergistic innovations in the future of OC treatment. Key areas of focus include overcoming resistance mechanisms, advancing precision stratification technologies, and optimizing global health strategies. The following sections delve into their potential and challenges within these domains (Figure 4).

### 5.1 Synergistic immune-angiogenesis modulation

The combination strategy of Bevacizumab with immunomodulators leverages the interaction between angiogenesis and the TME. Bevacizumab inhibits the VEGF signaling pathway, reducing regulatory T cell infiltration while promoting dendritic cell maturation, thus enhancing antigen presentation efficiency. This forms a synergistic foundation for immune checkpoint inhibitors such as anti-PD-1/PD-L1 therapies (Napoletano et al., 2019; Ribatti, 2022). The IMagyn050 trial demonstrated that the combination of atezolizumab and Bevacizumab increased the ORR to 35% in PD-L1 positive or high TMB patients, but PFS did not show a significant improvement in the overall population, underscoring the need for biomarker-based patient stratification to identify benefiting subgroups (Moore et al., 2021; Pignata et al., 2023). Further subgroup analysis from the PAOLA-1 trial revealed that Bevacizumab combined with Olaparib upregulated the interferon-γ signaling pathway and increased the formation of tertiary lymphoid structures within tumors (proportion increased by 40%), a mechanism linked to elevated MHC class I molecule expression (Harter et al., 2022; Ray-Coquard et al., 2023). Clinical trials targeting the conversion of “cold tumors” into “hot tumors” aim to reverse the immunosuppressive state of the TME. The GARNET trial found that FGFR inhibitors co-loaded with Bevacizumab in nanoparticles could simultaneously inhibit alternative angiogenic pathways and reduce the recruitment of MDSCs (with a 30% reduction), thereby remodeling the immune microenvironment (Rosario et al., 2024; Bhattacharya et al., 2023). Similarly, the INOVA trial demonstrated that the combination of Sintilimab (anti-PD-1) and Bevacizumab achieved an ORR of 48% in recurrent ovarian clear cell carcinoma, with tumor-infiltrating lymphocyte (TIL) density positively correlating with response rate (Peng et al., 2024). In murine models, combining personalized neoantigen vaccines with Bevacizumab increased the complete tumor remission rate from 20% (monotherapy) to 60%; however, clinical translation faces challenges such as antigen heterogeneity and delivery efficiency (Tanyi et al., 2018). Future research should integrate multi-omics approaches, including spatial transcriptomics and metabolomics, to dynamically track the evolution of TME. Moreover, the development of bispecific antibodies or engineered cell therapies may further enhance synergistic effects (Rosario et al., 2024; Landen et al., 2023). Prospective validation of stratification models based on PD-L1 expression, HRD, and TIL density will be critical to optimizing patient selection and minimizing immune-related adverse events (Moore et al., 2021; Pignata et al., 2023).

### 5.2 Multi-targeted approaches to overcome resistance

Bevacizumab, a key anti-angiogenic therapeutic agent, often faces limited clinical benefits due to adaptive remodeling of the TME and compensatory activation of signaling pathways. Overcoming resistance necessitates multidimensional strategies. In recent years, dual targeting of angiogenic pathways has gained significant attention. For example, targeting VEGF alone may become ineffective due to compensatory upregulation of other pro-angiogenic factors, such as Angiopoietin-2 (Ang-2) (Mitamura et al., 2018). Ang-2 promotes vascular remodeling and endothelial cell survival by binding to the Tie2 receptor. Inhibiting Ang-2 can synergistically block VEGF-independent angiogenic pathways, thereby delaying resistance progression (Deng et al., 2016). Preclinical models have demonstrated that simultaneous inhibition of VEGFR and Ang-2 significantly reduces tumor vascular density and improves microenvironment normalization, underscoring the potential of a dual-targeting approach (Al Wadi and Ghatage, 2016; Zhang X. et al., 2024). The hypoxic microenvironment also plays a pivotal role in driving resistance to anti-angiogenic therapy. HIF-1α, a central regulator of the hypoxia response, activates VEGF-independent angiogenic pathways and promotes metabolic reprogramming in tumor cells (Yue and Lu, 2023). For instance, HIF-1α enhances the survival capacity of tumor cells under hypoxic conditions by upregulating molecules such as carbonic anhydrase IX, while also inducing the expression of VEGF family members like placental growth factor (PlGF), which diminishes Bevacizumab efficacy (Boso et al., 2023). Inhibiting HIF-1α or its downstream effectors could potentially reverse hypoxia-mediated resistance phenotypes. Preclinical studies suggest that HIF-1α inhibitors reduce glycolytic activity and restore vascular normalization by downregulating VEGF-independent angiogenic pathways, such as PDGF/IL-8, thereby enhancing sensitivity to Bevacizumab (Deng et al., 2016). Furthermore, combining hypoxia regulators with Bevacizumab may improve therapeutic efficacy by suppressing tumor stem-like cell expansion and extracellular matrix remodeling (Olejarz et al., 2020). However, the specificity and toxicity of HIF-1α inhibitors require further optimization to avoid disrupting normal tissue oxygen homeostasis. Future research should employ multi-omics technologies to precisely identify resistance subtypes and develop sequential combination therapy strategies. For example, initiating treatment with Bevacizumab combined with an Ang-2 inhibitor, followed by HIF-1α-targeted agents upon disease progression, could enable dynamic intervention. Additionally, the development and application of traditional Chinese medicine (TCM) resources offer new avenues for overcoming Bevacizumab resistance. The multi-target regulatory effects of natural products provide an innovative direction for enhancing treatment efficacy (Wang X. et al., 2023). For instance, Bizzaro et al. found that a dose-dense regimen of DDP/PTX plus Bevacizumab was most effective in delaying tumor progression in preclinical models (Bizzaro et al., 2018). Active components of TCM, such as triptolide, can modulate the polarization states of TAMs and MDSCs, improving the immunosuppressive microenvironment (Bao et al., 2024). Curcumin, another anti-tumor component of TCM, inhibits tumor growth and angiogenesis through multiple mechanisms, including the PI3K/Akt, Wnt/β-catenin, JAK/STAT3, and MEK/ERK1/2 pathways, showing potential as an effective treatment for OC (Mohamadian et al., 2022). These findings suggest that the multidimensional “vascular-metabolism-immunity” regulatory capabilities of natural products could overcome the limitations of single-target therapies. However, further validation is required for clinical translation (Zhang et al., 2015).

### 5.3 Innovative drug delivery systems

Recent advances in nanotechnology-based Bevacizumab delivery systems have significantly improved the targeting specificity and safety of OC treatment. Nanoparticle carriers offer distinct advantages by optimizing drug release kinetics and enhancing tumor penetration (Zhang et al., 2015; Long et al., 2024). For example, the albumin-bound paclitaxel/Bevacizumab co-delivery system (AB160) has achieved a 54% overall response rate (ORR) in platinum-resistant patients, while reducing neutropenia incidence by 25%. This effect is potentially linked to albumin-mediated tumor-specific uptake and sustained release (Kalogera et al., 2024). Mesoporous silica nanoparticles modified with antibody fragments improve Bevacizumab accumulation in tumor tissues, resulting in a 2.8-fold increase in drug concentration in peritoneal metastases compared to conventional intravenous administration, while also mitigating the risk of proteinuria due to non-target organ exposure (Zhang et al., 2015). Furthermore, preclinical studies demonstrate that Bevacizumab-based nanomedicine enhances the intratumoral distribution of chemotherapeutic agents by inhibiting abnormal angiogenesis, thereby increasing paclitaxel’s antitumor efficacy with a 35% reduction in tumor volume (Cesca et al., 2016). The exploration of localized delivery strategies presents promising approaches for minimizing systemic toxicity. Intraperitoneal administration, which directly exposes tumor lesions to high drug concentrations, has shown survival benefits in platinum-resistant patients. Preliminary studies on pressurized intraperitoneal aerosol chemotherapy (PIPAC) combined with Bevacizumab indicate a median PFS extension of 3.2 months (Bakrin et al., 2018). However, the application of intraperitoneal Bevacizumab requires careful balancing of efficacy and safety. Retrospective analyses suggest an increased risk of gastrointestinal perforation, underscoring the importance of rigorous patient selection and optimized dosing regimens (Ueda et al., 2022). Notably, OC organoid-based nano-delivery systems, such as folate receptor-targeted liposomes, exhibit specific accumulation in peritoneal metastases, achieving a peritoneal drug concentration 3.5 times higher than in the bloodstream, thus offering an effective, low-toxicity solution for managing malignant ascites (Zhang et al., 2015; Singh et al., 2020). Future research should further assess the long-term safety of novel delivery systems and incorporate multimodal imaging technologies for real-time monitoring of drug distribution, facilitating precise dose regulation and optimized administration schedules.

### 5.4 Biomarker-driven precision medicine

In recent years, biomarker-driven personalized treatment strategies have significantly improved the clinical efficacy of Bevacizumab in OC (Werner et al., 2024). Dynamic monitoring of ctDNA facilitates real-time assessment of tumor burden and molecular heterogeneity. For instance, elevated plasma VEGF-C levels correlate negatively with the efficacy of Bevacizumab maintenance therapy, while ctDNA clearance rate can serve as an early predictor of treatment response (Ding et al., 2022; Liu et al., 2025b). An immune microenvironment analysis has revealed a correlation between the proportion of PD-1^+^ regulatory T cells and Bevacizumab resistance. Conversely, patients with high Ang-2 expression demonstrate a 2.3-fold increase in response rate to Bevacizumab, indicating that the interplay between immune-suppressive phenotypes and angiogenesis pathways may be a critical target for combination therapy (Park et al., 2023; Volk et al., 2023; Zhang H. et al., 2024). The integration of angiogenic gene signatures has further refined patient stratification, with the KELIM score—based on chemotherapy sensitivity models and VEGF receptor gene polymorphisms—providing an accurate prediction of Bevacizumab-induced PFS prolongation. Additionally, low AKAP12 expression is strongly associated with resistance to anti-VEGF therapies (You et al., 2022; Liang et al., 2022). The combination of multi-omics data has introduced new dimensions for dynamic monitoring. The whole-exome sequencing coupled with transcriptome analysis has revealed that abnormal activation of the CXCL12/CXCR4 axis in HRD^+^ tumors diminishes Bevacizumab efficacy, while metabolomic markers, such as LDH levels, are closely associated with the degree of Bevacizumab-induced vascular normalization (Liu X. et al., 2021; D'Alterio et al., 2022). Moreover, spatial multi-omics technologies, including multiplex immunofluorescence, enable the quantification of CD8^+^ T cell distribution and vascular density within the TME. The combined scoring approach significantly enhances predictive performance over single biomarkers (Wang et al., 2023c; Wang et al., 2022; Zhang et al., 2021; Hu et al., 2015). Notably, epigenetic regulation, such as pathogen-mimicry signaling mediated by ZNFX1, may influence Bevacizumab sensitivity by reshaping endothelial cell phenotypes, offering a novel target for overcoming resistance (Stojanovic et al., 2025). Future research should focus on the prospective validation of current biomarkers for clinical implementation and the development of AI-driven multimodal integration models, such as radiomics combined with liquid biopsy, to address challenges related to tumor heterogeneity and spatiotemporal evolution (Wang et al., 2023d; Xu et al., 2024).

## 6 Conclusion

Bevacizumab inhibits tumor angiogenesis and remodels the immunosuppressive TME by targeting VEGF-A, becoming a core drug in the comprehensive treatment of OC. Its combination with chemotherapy, PARP inhibitors, or immune checkpoint inhibitors can significantly prolong patient PFS, especially in patients with HRD positivity. Current trends in combination therapy concentrate on dual-target strategies, such as VEGF/Ang-2 bispecific antibodies, synergistic immunotherapy-anti-angiogenesis treatments, and intelligent nano-delivery systems. Meanwhile, precision stratification strategies based on biomarkers are progressively advancing. However, the OS benefit is limited, and the mechanisms of resistance, including alternative pathway activation and hypoxia-driven malignant transformation, as well as the lack of effective predictive biomarkers, remain major challenges. Treatment toxicity, including hypertension and proteinuria, along with high costs, further limits its application. Future efforts should focus on optimizing drug delivery systems, such as targeted nanocarriers, exploring multi-target inhibitors to overcome resistance, and conducting prospective clinical trials to validate new combination regimens. This should involve integrating artificial intelligence with dynamic biomarker monitoring to achieve personalized treatment.
